# Simple Treatment of Prolapsus Ani

**Published:** 1847-09

**Authors:** 


*Simple Treatment of Prolapsus Ani.* By Dr. Hake.—Take a piece of sponge four or five inches long, an inch and a half wide, and half an inch thick—the more elastic a bit you can find the better; roll this, in a damp but not wet state, pretty tightly, so that the roll, if relaxed, would be ready to spring back into its full length, and it will then make a roll of some little substance, round, but still soft; and its length, when thus rolled, will of course be an inch and a half. Apply it then lengthwise to the anus, so that it may be pressed, about the centre of it, quite home and firmly to that part. Taking care that it may remain so, stretch a length of adhesive plaster, about fourteen inches long, and three and a half wide, more or less, straight across the nates, rather low down, and contrive so that while the plaster adheres on one side, you press the other side closer to its opposite before you fix the length finally where it is to remain. Then sit down, at first gently upon it, and it will become very firm and fast as long as the plaster is good. I need not say that these two pressures constantly going on do the work capitally, and without any inconvenience worth speaking of—I mean, the two pressures of the roll of sponge always striving to unwrap itself, and the cross-band of adhesive plaster always keeping it from doing so by holding the nates sufficiently close together to hinder it. The working is really perfect when a little use and management has got a person into the way of it. But to facilitate matters, I will set down a few observations, at the risk of being tedious and more particular than I need be.

I never put this on until that time of day when I am going to be standing about, or to take exercise, whether walking, riding, or driving; but it should be put on then for all of these. In the evening, I take off the plaster, but leave the sponge in its place, where it has got by that time so firmly fixed by gradual spreading and swelling, that there is no danger that anything short of a great exertion will loosen it, and it is of course more comfortable to do without the plaster when it is not wanted. The sponge should be washed in cold water every time it is taken off, and in cold weather the plaster should just cross the fire before it is put on; in moderately warm weather it will adhere of itself, especially if it is sat upon for half a minute. The same plaster is better the second day than even the first, and will do very well the third day—this where economy is an object.

Wash the parts where the plaster goes every morning, or oftener, with cold water, or water and vinegar, wash them well, and the skin will never suffer.

If the plaster leaves something sticky behind it when it is taken off, rub it with a very little spirit of wine, and the towel will remove it.

If there be an irritation about the anus, or gut that comes down, wash it with vinegar and water, and the relief will be wonderful, and that part of the evil soon cured. This wash cannot be too much praised for this purpose, for piles, and for the like. I leave it for you to say whether something might not be dropped upon the sponge, or the sponge dipped in something which would promote a complete cure. What I have said is perfectly cleanly, secures exercise and comfort, and very gradually, I believe, tends to set things right again. The relief is, indeed, so perfect, and it is relief from such suffering, that, without a bit of braggadocio, I do think no sufferer from such malady would feel that he could be grateful enough for being brought acquainted with the treatment I have described, though its perfect management will require a little experience at least, and perhaps some advice at first.—*Ibid.*

